# Stable and Fast Deep Mutual Information Maximization Based on Wasserstein Distance

**DOI:** 10.3390/e25121607

**Published:** 2023-11-30

**Authors:** Xing He, Changgen Peng, Lin Wang, Weijie Tan, Zifan Wang

**Affiliations:** 1 State Key Laboratory of Public Big Data, College of Computer Science and Technology, Guizhou University, Guiyang 550025, China; gs.hex20@gzu.edu.cn; 2 Guizhou Key Laboratory of Pattern Recognition and Intelligent System, Guizhou Minzu University, Guiyang 550025, China; wanglin@gzmu.edu.cn; 3 Guizhou Big Data Academy, Guizhou University, Guiyang 550025, China; wjtan@gzu.edu.cn; 4 Institute of Guizhou Aerospace Measuring and Testing Technology, Guiyang 550025, China; wzf1997@foxmail.com

**Keywords:** machine learning, deep learning, unsupervised learning, encoder network, mutual information estimation

## Abstract

Deep learning is one of the most exciting and promising techniques in the field of artificial intelligence (AI), which drives AI applications to be more intelligent and comprehensive. However, existing deep learning techniques usually require a large amount of expensive labeled data, which limit the application and development of deep learning techniques, and thus it is imperative to study unsupervised machine learning. The learning of deep representations by mutual information estimation and maximization (Deep InfoMax or DIM) method has achieved unprecedented results in the field of unsupervised learning. However, in the DIM method, to restrict the encoder to learn more normalized feature representations, an adversarial network learning method is used to make the encoder output consistent with a priori positively distributed data. As we know, the model training of the adversarial network learning method is difficult to converge, because there is a logarithmic function in the loss function of the cross-entropy measure, and the gradient of the model parameters is susceptible to the “gradient explosion” or “gradient disappearance” phenomena, which makes the training of the DIM method extremely unstable. In this regard, we propose a Wasserstein distance-based DIM method to solve the stability problem of model training, and our method is called the WDIM. Subsequently, the training stability of the WDIM method and the classification ability of unsupervised learning are verified on the CIFAR10, CIFAR100, and STL10 datasets. The experiments show that our proposed WDIM method is more stable to parameter updates, has faster model convergence, and at the same time, has almost the same accuracy as the DIM method on the classification task of unsupervised learning. Finally, we also propose a reflection of future research for the WDIM method, aiming to provide a research idea and direction for solving the image classification task with unsupervised learning.

## 1. Introduction

Unsupervised learning is a machine learning (ML) training method, which is essentially a statistical means to discover underlying structures or attributes on unlabeled datasets. Since unsupervised learning methods have the advantage of training networks without labeled data, it appears to be crucial for large-scale data collection and is of great importance to facilitate the development of artificial intelligence.

In the past, the main unsupervised learning algorithms have been principal component analysis methods [1], isometric mapping methods [2], locally linear embedding methods [3], Laplace feature mapping methods [4], Hesse local linear embedding methods [5], and local tangent space alignment methods [6]. However, for the high-dimensional data case, all these methods have some limitations. In recent years, the application of unsupervised learning has achieved some success. the Generative Adversarial Networks (GANs) network proposed by Goodfellow et al. [7] has achieved excellent results in the field of image generation, and many research scholars have proposed variants of GAN networks based on this [8], and these frameworks provide sample generation for unsupervised learning as well as theoretical guidance for feature encoding and decoding [9]. Despite the striking early successes in unsupervised representation learning using GANs, they have since been superseded by self-supervision-based approaches.

At this stage, to the best of our knowledge, many unsupervised learning methods train feature extractors by maximizing the Mutual Information (MI) between different views, and these methods are rapidly closing the gap with supervised methods. The literature [10] proposes the Deep InfoMax (DIM) method, which performs unsupervised representation learning by maximizing the mutual information between the input and the output of the deep neural network encoder. However, to solve the problem of estimating the MI, the literature [11] proposed the Mutual Information Neural Network Estimator (MINE). In some applications, MINE can be used to implement the training of GANs and to achieve information bottlenecks, as well as supervised classification [12], among others. Based on this, it has been argued that the success of MINE methods is not only attributed to the properties of MI; they depend heavily on the structural choice of the feature extractor and the parametric bias of the MI estimator employed [13]. The momentum contrast (MoCo) proposed by He et al. [14] builds a dynamic dictionary with queues and moving average encoders, thus facilitating contrastive unsupervised learning. Some researchers have also validated its effectiveness by implementing a modification of SimCLR [15] in the MoCo framework, which outperforms SimCLR and does not require large sample training [16]. Han et al. [17] proposed a novel self-supervised co-training method to improve the popular infoNCE loss [18], and improved the model convergence speed.

Although the DIM method has achieved excellent results in classification tasks with unsupervised learning, the party uses a cross-entropy-based method to measure the distance between the encoder’s output and the a priori positive-earthly distribution, and is trained using the adversarial training method, which brings the encoder’s output closer to the positive-earthly distribution, thus making the encoder’s output more regular. It is well known that due to the presence of a logarithmic function in the loss function of the cross-entropy measure, the learning of adversarial network is very unstable. This is because, when calculating the gradient of the model parameters, it is very easy to have the phenomena of “gradient explosion” and “gradient disappearance”, which is the reason why the DIM method is extremely unstable in the training of the model. Meanwhile, it is difficult to reach the Nash equilibrium point of model convergence for adversarial training, which is a critical problem that is difficult to solve in generative adversarial networks.

To address the instability of the DIM method in training the model, first, we adopt the difference between the output of the Wasserstein distance metric encoder and the prior distribution as the loss of the prior discriminator based on the superiority of the Wasserstein distance measure of the difference between the two high-dimensional random variables, and this metric makes the training of prior discriminative networks more stable and the model convergence faster. Secondly, for the training of the a priori discriminant network in the DIM method, we do not need to use the adversarial training method to train the sub-network, we directly use the loss value of the Wasserstein distance metric to calculate the gradient of the parameters of the sub-network, and further training on the update of the model parameters can be performed so that there is a breakthrough in this improvement and the performance of the model is significant. Our proposed WDIM method is validated on CIFAR10, CIFAR100, and STL10 datasets. The experiments show that the WDIM method is more stable for model training and faster for model convergence. The main contributions of this paper are as follows:

1. To propose a method based on the Wasserstein distance metric to measure the difference between the output of the encoder and the a priori positive terrestrial distribution, which is used as the loss of the a priori discriminator network.
2. To adopt the method of the optimal transport path to estimate the Wasserstein distance, which is not the same as the method of model parameter tailoring to estimate the Wasserstein distance, and the experiments show that such an estimation computation is more capable of reflecting the advantages of the Wasserstein distance, and the estimated distance is more accurate and reliable.
3. For the training of the a priori discriminative network in the DIM method, we do not need to use the method of adversarial training, we only need to minimize the distance between the output of the coding network and the a priori positive distribution to achieve the training of the a priori discriminative network, which makes the training of the model more efficient, and the stability of the convergence of the model is higher.

The article describes the research work closely related to this paper in Section 2, the DIM methodology in Section 3, the theory of the WDIM methodology in Section 4, the experiments with the methodology in Section 5, and the conclusions and outlook in Section 6.

## 2. Related Works

While supervised learning has made tremendous progress in the application of machine learning systems, unsupervised learning has not been as widely popularized and it remains an important and challenging endeavor in artificial intelligence. Unsupervised learning methods have the advantage of not requiring expensive labeled data to train networks, which appears to be crucial for successfully collecting today’s large amount of visual data, which are of great significance in promoting the development of AI. However, the performance metrics of unsupervised networks have been lagging behind supervised networks in practical applications, especially in the field of large-scale visual data recognition. To narrow the performance gap between unsupervised learning and supervised learning methods, many researchers and scholars have devoted themselves to unsupervised network learning methods, and excellent research results have been achieved at this stage. Unsupervised learning can be broadly categorized into unsupervised learning methods based on feature encoding networks and unsupervised learning methods based on clustering algorithms according to the differences in model learning methods.

Unsupervised learning methods based on feature encoding networks: many recent unsupervised or self-supervised representation learning methods use the structure or properties of the data themselves to automatically generate labels or features for model training. By maximizing the mutual information (MI) between different views to train feature extractors, these methods are rapidly closing the gap with supervised methods [19]. The autoencoders proposed in the literature [20] are a direct modification of the traditional autoencoder structure, forming sub-networks for unsupervised representation learning. In 2018, Deep InfoMax (DIM) proposed in the literature [10] is the most popular unsupervised representation learning method. Unsupervised representation learning is performed by maximizing the mutual information between the input and the output of the deep neural network encoder. Based on the DIM method, Bachman et al. [21] proposed a method to maximize the mutual information between feature information for the high-level factors of multiple views of a shared environment. However, to address the problem of MI estimation, the literature [11] proposes the mutual information neural estimator (MINE), which is linearly scalable in dimensionality and sample size. In some applications, the MINE can be used to implement the training of GANs and be used for information bottlenecks and supervised classification [22]. Based on this, the literature [13,23] argues that the success of MINE methods is not only attributed to the properties of MI; they depend heavily on the choice of feature extractor structure and the parameterization bias of the employed MI estimator. He et al. [14] proposed momentum contrast (MoCo) for unsupervised learning of visual representations. The method builds a dynamic dictionary with queues and moving average encoders, thus facilitating contrasted unsupervised learning, and the representations learned by MoCo can be well transferred to downstream tasks. The literature [24] verified its effectiveness by implementing a modification of SimCLR in the MoCo framework, which outperforms SimCLR and does not require large training batches. Han et al. [17] proposed a novel self-supervised co-training method to improve the popular infoNCE loss, and the proposed method has comparable performance to other self-supervised methods, while the training efficiency significantly improved.

Unsupervised learning methods based on clustering algorithms: Deep Clustering, proposed by Caron et al. [25], is a clustering method that jointly learns neural network parameters and the resulting feature clustering assignments and outperforms the current state-of-the-art by a significant margin on all standard benchmarks. The literature [26] describes a method for training embedding functions to maximize the local aggregation metric, allowing similar data instances to move together in the embedding space while allowing dissimilar instances to separate. In neural network training, “smoothing the label/prediction distribution” has been shown to help prevent overconfidence in the model and is essential for learning more robust visual representations [27]. The literature [28] suggests learning image features by training convolutional networks (ConvNets) to be applied to recognize 2D rotations of input images. Wu et al. [29] train neural net classifiers on annotated category-labeled datasets to be extended to unsupervised learning environments and exceed the state-of-the-art by a large margin on the ImageNet dataset. The literature [30] introduces the generalized data transformation framework, a framework that allows the simultaneous injection of invariance and uniqueness into representations, and applies it to representation learning in unlabeled videos [31], as well as in recurrent neural networks, where there are also some excellent research results [32].

In recent years, to the best of our knowledge, the most dominant unsupervised learning method, DIM [10], is one of the most effective unsupervised learning methods based on feature encoding networks. DIM performs unsupervised representation learning by maximizing the mutual information between the output of the hidden layer of the encoder and the output of the encoder, a process referred to as global mutual information maximization. At the same time, maximizing the location information of the feature map of the hidden layer and the location information of the encoded vector of the output incorporates the location knowledge from the input into the target, a process called local mutual information maximization. Thus, the representational capability of the downstream task is substantially improved. The features of the representation are further controlled by matching the adversarial with the prior distribution. This method outperforms some popular unsupervised learning methods, and DIM opens up new avenues for unsupervised learning of representations. Currently, the research on DIM-based variant methods [21,33] has become one of the hotter topics.

Although the DIM method achieves good unsupervised learning results, its model training process is extremely unstable; therefore, the technique of a gradient penalty on model parameters is used to make the model stable for training in the process of DIM training, which fundamentally limits the learning ability of the network and is not desirable. We conducted an in-depth study of the main reasons for the highly unstable training of the DIM method, and we found that the features of the representation are further controlled by matching the output of the encoder with the prior distribution in the adversarial learning approach in the DIM method. We know that the learning of adversarial networks is extremely unstable because of the presence of a logarithmic function in the loss function of the cross-entropy inscription, which makes it easy to calculate the gradient of the model parameters and the phenomenon of “gradient explosion”, which makes the training of the prior discriminative network part of the DIM model extremely unstable. Therefore, to characterize the loss function metric during the adversarial process, based on the superiority of the Wasserstein distance measure of the difference between two random variables [34], we propose the WDIM method, which can stabilize the training of the model well and the model converges faster during the training process.

**Figure 1:**
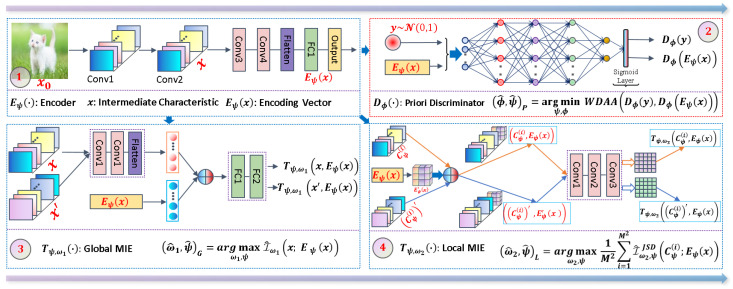
WDIM method model overview. Five main models are trained, which are the encoder $E_{\psi }$ for deep feature learning, the prior discriminator $D_{\phi }$, the global mutual information estimation network $T_{\psi ,\omega_{1}}$, and the local mutual information estimation network $T_{\psi ,\omega_{2}}$.

The execution process of our proposed WDIM method is shown in Figure 1. Four main sub-network models are trained in the WDIM method. The sub-network used for feature extraction is called $E_{\psi }$, also known as the encoder network. The a priori discriminative network $D_{\phi }$ is the second sub-network, which takes the output of the feature extraction network $E_{\psi }$ as the input, and hopes that the predicted output has the same distribution as the a priori’s normal distribution, and its main purpose is to make the features extracted by $E_{\psi }$ more normalized so that it is easy to implement the subsequent classification tasks. The third sub-network is the global mutual information estimation network $T_{\psi ,\omega_{1}}$, and we hope that the output features of $E_{\psi }$ can contain more information about the more global horizons of the inputs of $E_{\psi }$. In the same principle, the fourth sub-network $T_{\psi ,\omega_{2}}$, to get the output features of $E_{\psi }$ to include more position information of the input of $E_{\psi }$, incorporates the knowledge of the position in the input into the objective, and learns more detailed information such as local invariance of the input.

## 3. DIM Method

Our WDIM method is improved on top of the DIM method. Therefore, in this subsection, we first give the construction procedure of the loss function of the DIM method and qualitatively analyze the main reasons for the instability of the training model of the DIM method in the next section.

### 3.1. Loss Function of DIM Method

In the DIM method, the optimal representation of the feature extraction network $E_{\Psi }\left(\cdot \right)$ on the training data is obtained by optimizing the loss function Equation (6). In the DIM method, the lower bound of mutual information is expressed as follows through the Donsker–Varadhan representation [35]:

(1) $$I\left(X,Y\right):=D_{KL}\left(J∥M\right)\geq {\hat{I}}_{\omega }^{\left(DV\right)}\left(X,Y\right):=E_{J}\left[T_{\omega }\left(x,y\right)\right]-\log E_{M}\left[e^{T_{\omega }\left(x,y\right)}\right]$$

where the random variables *X* and *Y* denote the training dataset, and the encoding vector set, respectively. In particular, note that *X* is used as the middle layer feature of the encoder in achieving the approximate estimation of the mutual information. I(X,Y) denotes the mutual information between the random variables *X* and *Y*, J denotes the joint distribution function of *X* and *Y*, M denotes the product of the edge distribution functions of *X* and *Y*, and $D_{KL}\left(J∥M\right)$ denotes the KL−divergence between J and M. $T_{\omega }\left(x,y\right)$ denotes the neural network with (x,y) sample pairs as the input and ω as the parameter, and ${\hat{I}}_{\omega }^{\left(DV\right)}\left(X,Y\right)$ denotes the lower bound of the Donsker–Varadhan representation of the mutual information between *X* and *Y*.

For the representation of global mutual information estimation, this is achieved by optimizing the following function:

(2) $${\left({\hat{\omega }}_{1},\hat{\psi }\right)}_{G}=\underset{\omega_{1},\psi }{\arg\max}{\hat{I}}_{\omega_{1}}\left(X;E_{\psi }\left(X\right)\right)$$

where $E_{\psi }\left(X\right)$ denotes the encoding vector of the encoder output with input *X* and parameter ψ, ${\hat{I}}_{\omega_{1}}\left(\cdot \right)$ denotes the global mutual information estimator with parameter $\omega_{1}$, and the global mutual information maximization is denoted by ${\left({\hat{\omega }}_{1},\hat{\psi }\right)}_{G}$. In the process of optimization, the maximization estimate of ${\hat{I}}_{\omega }^{\left(DV\right)}\left(X,Y\right)$ is rewritten as Jensen−Shannon divergence (JSD) representation with an upper bound since $D_{KL}\left(∥∥M\right)$ has no upper exact bound.

(3) $${\hat{I}}_{\omega ,\psi }^{JSD}\left(X;E_{\psi }\left(X\right)\right):=E_{P}\left[-sp\left(-T_{\psi ,\omega }\left(x,E_{\psi }\left(x\right)\right)\right)\right]-E_{P\times \tilde{P}}\left[sp\left(T_{\psi ,\omega }\left(x^{\prime },E_{\psi }\left(x\right)\right)\right)\right]$$

where sp(·) denotes the softplus function and $sp\left(z\right)=\log\left(1+e^{z}\right)$. $\left(x,E_{\psi }\left(x\right)\right)$ denotes the positive sample pair and $\left(x^{\prime },E_{\psi }\left(x\right)\right)$ denotes the negative sample pair. P denotes the probability distribution of the random variable *X*, and $\tilde{P}$ denotes the probability distribution of the negative sample.

Based on the representation of global mutual information estimation, the same representation of local mutual information estimation can be obtained.

(4) $${\left({\hat{\omega }}_{2},\hat{\psi }\right)}_{L}=\underset{\omega_{2},\psi }{\arg\max}\frac{1}{M^{2}}\sum_{i=1}^{M^{2}}{\hat{I}}_{\omega_{2},\psi }^{JSD}\left(C_{\psi }^{\left(i\right)};E_{\psi }\left(X\right)\right)$$

where $M^{2}$ denotes the number of features of a certain hidden layer, $C_{\psi }^{\left(i\right)}$ denotes the i-th feature of the hidden layer, ${\hat{I}}_{\omega_{2},\psi }^{JSD}\left(\cdot \right)$ denotes the local mutual information estimator with parameter $\omega_{2}$, and the local mutual information maximization is denoted by ${\left({\hat{\omega }}_{2},\hat{\psi }\right)}_{L}$.

In variational self-encoders [36], it is more desirable that the encoded vectors obey a priori the standard normal distribution, which is beneficial to make the encoding space more regular and even to decouple features for subsequent learning. Therefore, the DIM algorithm also wants to add this constraint, only, here, the adversarial regularized representation is used.

(5) $$\begin{array}{cc} {\left(\hat{\phi },\hat{\psi }\right)}_{P}\; & =\underset{\psi }{\arg\min}\;\underset{\phi }{\arg\max}{\hat{D}}_{\phi }\left(V∥U_{\psi ,P}\right)=E_{V}\left[\log D_{\phi }\left(y\right)\right]+E_{P}\left[\log\left(1-D_{\phi }\left(E_{\psi }\left(x\right)\right)\right)\right] \end{array}$$

where V denotes the standardized normal distribution and $D_{\phi }$ is a neural network discriminator with parameter ϕ. $U_{\psi ,P}$ denotes the edge distribution that pushes the samples from distribution P. The full loss of the optimization objective of the DIM method is the weighted sum of the three loss functions of Equations (2), (4), and (5), as follows:

(6) $$\begin{array}{cc} \;\mathrm{Loos}\;\; & =\underset{\omega_{1},\omega_{2},\psi ,\phi }{\arg\max}\left(\alpha {\hat{I}}_{\omega_{1},\psi }^{JSD}\left(X;E_{\psi }\left(X\right)\right)+\frac{\beta }{M^{2}}\sum_{i=1}^{M^{2}}{\hat{I}}_{\omega_{2},\psi }\left(X^{i};E_{\psi }\left(X\right)\right)\right)\\ \; & \;+\underset{\psi }{\arg\min}\;\underset{\phi }{\arg\max}\gamma {\hat{D}}_{\phi }\left(V∥U_{\psi ,P}\right) \end{array}$$

where α denotes the weight of the global mutual information loss term, β denotes the weight of the local mutual information loss term, and γ denotes the weight of the a priori loss term. In the objective function of the DIM algorithm, Equation (6), the third term ${\left(\hat{\phi },\hat{\psi }\right)}_{P}$ contains a log(·) function, which makes the adversarial training suffer from a serious “gradient explosion” phenomenon. To be able to dissect this phenomenon qualitatively, we will quantitatively analyze the root cause of the instability of the training model in the DIM method in the next section.

### 3.2. Stability Analysis of DIM Method

In this section, we analyze in detail the main reasons for the instability of the model trained by the DIM method, which employs Equation (5) to measure the distance between the a priori n-tai distribution and the feature vector as the loss of the a priori discriminator, at which point there are a series of problems with the stability of the model training. Equation (5) is essentially a cross-entropy loss, and the purpose of optimizing the loss function is to hope that the true sample $D_{\phi }\left(y\right)\to 1$ and the false sample $D_{\phi }\left(E_{\psi }\left(x\right)\right)\to 0$ so that the output of the feature extraction network $E_{\psi }\left(x\right)$ obeys the a priori nontrivial distribution V. Subsequently, the a priori discriminative network is trained using the adversarial training method, and the adversarial training method suffers from “gradient explosion” or “gradient vanishing" phenomena [37].

**Figure 2:**
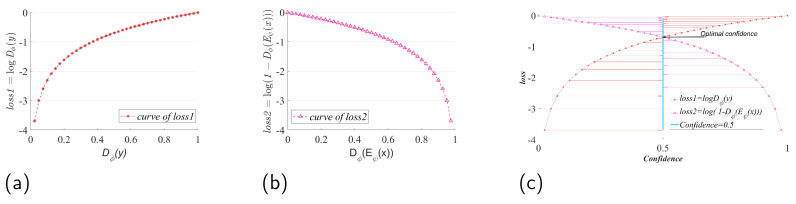
Quantitative analysis of the stability of the training model for the DIM method.

Gradient explosion: as shown in Figure 2, in (a), when $D_{\phi }\left(y\right)\to 0$, loss→−∞, there is a “gradient explosion” phenomenon, which leads to the failure of model training to converge. In (b), when $D_{\phi }\left(E_{\psi }\left(x\right)\right)\to 0$, the loss→−∞, which also leads to the phenomenon of “gradient explosion”, which then leads to the failure of model training convergence. In (c), the optimal confidence obtained by optimizing Equation (5) is theoretically found at the intersection of loss1, loss2, and Confidence. However, since the alternating training model may not be able to find the Nash equilibrium point, we urgently need to reconstruct a more stable optimization function to replace Equation (5), so as to ensure that the training model of the DIM method can provide a stable gradient computation, as well as to ensure that the training process will not appear as the phenomenon of a “gradient explosion”.

Gradient vanishing: the most common methods to measure the difference between two distributions are KL−divergence and J−divergence. However, there are still serious problems with the distances measured by these methods. For one, for any two probability distributions P(x) and Q(y), since $D_{KL}\left(P∥Q\right)\neq D_{KL}\left(Q∥P\right)$, the distance measured by KL−divergence does not satisfy the definition of distance in a practical sense. To solve the asymmetry problem of KL−divergence, JS−divergence with symmetric property is proposed to measure the distance between two distributions, so the KL−divergence measure is discarded in many machine learning algorithms and the JS−divergence measure is adopted instead. Second, in terms of the range of values of distance, $D_{JS}\left(P∥Q\right)\in \left[0,\log2\right]$, when P(x)=Q(y), $D_{JS}\left(P∥Q\right)=0$. Therefore, theoretically speaking, JS−divergence is indeed a feasible method to measure the distance between two distributions. However, this is not the practice case as the dimensional catastrophe problem caused by multidimensional data makes the distribution P(x) and the distribution Q(y) not always have overlapping regions in the probability density space, $D_{JS}\left(P∥Q\right)\equiv \log2$. This is fatal to the use of stochastic gradient descent to optimize the objective function because the gradient computation provided by the objective function with constant log2 is equal to 0, which leads to the phenomenon of “gradient disappearance” and, therefore, the model parameters cannot be updated.

Through the above analysis, there are “gradient explosion” and “gradient disappearance” phenomena in the process of training models by the DIM method, and it is difficult to converge the adversarial training model to the “Nash equilibrium”. For this reason, we need to improve Equation (5) so that the training model can converge stably, and at the same time, discard the loss function of the cross-entropy measure, which we find is unnecessary. In the next subsection, we describe our proposed WDIM method in detail.

## 4. WDIM Method

In this subsection, we focus on two objectives. First, we propose an improvement to Equation (5) based on the Wasserstein distance, which solves the “gradient explosion” phenomenon of training a priori discriminators. The second is to learn an encoder with good generalization ability and better characterization ability. We propose a decoupled learning method called intermediate layer features, which allows different filters to learn a more efficient class of feature representations, thus separating different types of features. Therefore, to lead to the computation of the distance between the output of the encoding vector and the output of the prior distribution used in our method, we first introduce the computation of the Wasserstein distance approximation estimate.

### 4.1. Approximate Estimation Method of Wasserstein Distance

To measure the difference between two distributions and, at the same time, to solve the “gradient disappearance” phenomenon of the JS−divergence measure distance, the method of estimating the distance between two distributions based on the Wasserstein distance is proposed [37], and we give the Wasserstein distance definition and its approximate optimal estimation algorithm.

Wasserstein distance definition: let Π(P,Q) be the set of all possible joint probability distributions for the combination of two probability distributions P(x) and Q(y), and for any joint probability distribution γ(x,y) in the set, the distance d(x,y) of the sample pair (x,y)∼γ distribution is defined as follows:

(7) $$W\left(P,Q\right)=\underset{\gamma ∼\Pi (P,Q)}{\inf}\;\int \int \;\gamma \left(x,y\right)d\left(x,y\right)=\underset{\gamma ∼\Pi (P,Q)}{\inf}E_{(x,y)∼\gamma }\left[d\left(x,y\right)\right]$$

where d(x,y) is the cost function. For each possible joint distribution γ, a sample *x* and *y* can be obtained by sampling (x,y)∼γ from it and calculating the cost d(x,y) between the pair, so the expectation $E_{(x,y)∼\gamma }\left[d\left(x,y\right)\right]$ of the sample to the cost under that joint distribution γ can be calculated. The lower bound that can be taken for the expectation value in all possible joint distributions is the Wasserstein distance.

In machine learning, it is very difficult to compute the distance between two distributions by sampling. Therefore, the Wasserstein distance needs to be approximated using the optimal transport path of Sinkhorn’s algorithm [38]. Sinkhorn’s algorithm encourages the transport of most of the low-traffic paths. Therefore, entropy regularization is introduced to penalize sparse paths, and the Wasserstein distance is further approximated using immobile point iterations to estimate the Wasserstein distance. In Algorithm 1, we give the motionless point approximation estimation algorithm for the Wasserstein distance, which we call WDAA.

In WDAA, $P_{\left(i,j\right)}=u_{i}K_{i,j}v_{j}$, ∀(i,j)∈[n]×[m],$P_{\left(i,j\right)}$ denotes the regularized Kantorovich problem, where $C_{i,j}$ denotes the cost of transferring a unit mass from $a_{i}$ to $b_{j}$, and (i,j) denotes the matrix subscript in the cost matrix *C*. $P=\mathrm{diag}\left(u\right)K\mathrm{diag}\left(v\right),\left(u,v\right)\in R_{+}^{n}\times$$R_{+}^{m},$$K_{i,j}=e^{-C_{i,j}/\varepsilon }$. ε is the regularization factor whose magnitude determines the strength of the regularization effect. And the vectors *u* and *v* are the variables to be required by Sinkhorn’s algorithm by adding the mass conservation conditions for optimal transport a=u⊙Kv and $b=v⊙\left(K^{T}v\right)$, where ⊙ is the Hadamard product of the vectors. Therefore, we can solve for *u*, *v* by iterative means solving for *u*, *v*. At each step, *u* is updated first, then *v* is updated, and eventually, the iterations converge, so the following iterative equation is obtained:

(8) $$u^{t+1}=\frac{a}{Kv^{t}}+u^{t},v^{t+1}=\frac{b}{K^{T}u^{t+1}}+v^{t}$$

**Figure 3:**
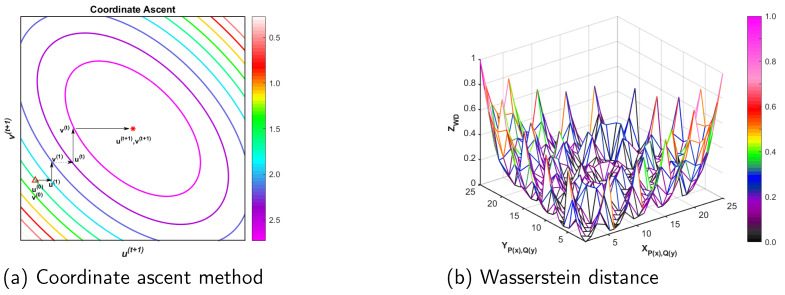
Visualization of the approximate solution of the Wasserstein distance between any two distributions P(x) and Q(y).

The Wasserstein distance approximation is solved using the optimal transport Sinkhorn algorithm, which, according to Equation (8), is essentially an iterative approximation using an immobile point equation, and the process of solving the immobile point equation is a Coordinate Ascend. In Figure 3a, we give the one-dimensional case, using the Coordinate Ascend method to solve $u^{t+1}$ and $v^{t+1}$ in the one-dimensional case. From the figure, we can see that, first, the variables $u^{0}$ and $v^{0}$ are initialized randomly. Second, $u^{1}$ is updated with $u^{0}$ and $v^{0}$ according to the first equation of Equation (8), and after obtaining $u^{1}$, $v^{1}$ is updated with $u^{1}$ according to the second equation of Equation (8). $u^{t}$ and $v^{t}$ are obtained after finitely alternating many iterations, $u^{t+1}$ is updated with $v^{t}$, $u^{t+1}$ to update $v^{t+1}$, and then we obtain $u^{t+1}$ and $v^{t+1}$, which are the optimal approximate solutions. Finally, using the $u^{t+1}$ and $v^{t+1}$ obtained from the above solution, the cost matrix *C* is updated to obtain the Wasserstein distance approximation to calculate the distance between any two distributions.
**Algorithm 1** Wasserstein distance approximation algorithm (WDAA)**Input:** Input any two probability distributions P(x), Q(y), the maximum number of iterations Max_iter, and the control threshold Err_thresh of the coordinate ascent method. 1: Initialization $u^{0}$, $v^{0}$ 2: C←Cost_Matrix(P(x),Q(y))    //Calculate the cost matrix *C* 3: $K\leftarrow e^{-C/\varepsilon }$ 4: $a\leftarrow u^{0}⊙Kv^{0}$,$b\leftarrow v^{0}⊙\left(K^{T}u^{0}\right)$ 5: t←0 6: **while** t<Max_iter **do** 7:     $u^{t+1}\leftarrow \frac{a}{Kv^{t}}+u^{t}$, $v^{t+1}\leftarrow \frac{b}{K^{T}u^{t+1}}+v^{t}$ 8:     **if** $sum\left(abs\left(u^{t+1}-u^{t}\right)\right)\leq Err\text{\_}thresh$ **then** 9:         $u^{*}\leftarrow u^{t+1}$10:         $v^{*}\leftarrow v^{t+1}$11:         break12:     **end if**13:     t←t+114: **end while**15: $W_{d}\leftarrow D\left(C,u^{*},v^{*}\right)$

In Figure 3b, we discretize the representation P(x) and Q(y) such that $X_{P(x),Q(x)}=\left(P_{i},Q_{j}\right),i,j\in \left[0,C\right]$, and $\left(P_{i},Q_{j}\right)$ is a pairwise two-dimensional vector taking values in [0,1] at a step of 0.25, which can represent our constructed two-dimensional distribution, where *C* denotes the total number of pairwise two-dimensional vectors. Using the definition principle of $X_{P(x),Q(x)}$, we can define $Y_{P(x),Q(x)}=X_{P(x),Q(x)}$ in the same way. With the above representations of $X_{P(x),Q(x)}$ and $Y_{P(x),Q(x)}$, we can represent the Wasserstein distance visualization between the two-dimensional probability distributions P(x) and Q(y) in the three-dimensional space. It can be seen that, firstly, the Wasserstein distance between the probability distributions P(x) and Q(y) obtains a maximum value of 1 when $\left(P_{i},Q_{j}\right)=\left(0,0\right)$ and $\left(Q_{j},P_{i}\right)=\left(1,1\right)$, or $\left(P_{i},Q_{j}\right)=\left(1,1\right)$ and $\left(Q_{j},P_{i}\right)=\left(0,0\right)$. Secondly, the Wasserstein distance between the probability distributions $X_{P(x),Q(x)}$ and $Y_{P(x),Q(x)}$ plane diagonally. When $X_{P(x),Q(x)}=Y_{P(x),Q(x)}$, the Wasserstein distance between the probability distributions P(x) and Q(y) obtains the minimum value 0. Thirdly, for other values of the probability distributions P(x) and Q(y), the Wasserstein distance between the probability distributions P(x) and Q(y) is obtained to belong between (0,1).

In order to approximate the Wasserstein distance $W_{d}$ between $E_{\psi }\left(x\right)$ and $D_{\phi }\left(E_{\psi }\left(x\right)\right)$ in Equation (5), the Wasserstein distance approximation algorithm is given in Algorithm 1. The Wasserstein distance has an upper-certainty bound on the measure of the distance between any two distributions when $E_{\psi }\left(x\right)\in \left[0,1\right]$ and $D_{\phi }\left(E_{\psi }\left(x\right)\right)\in$[0,1]. Therefore, the Wasserstein distance measure of the distance between any two distributions can avoid the “gradient explosion” phenomenon during the training of the model, and the Wasserstein distance measure of the distance between two distributions does not depend on whether the distributions have overlapping regions. The Wasserstein distance measure between two distributions does not depend on whether there is an overlap between the distributions. Therefore, we will improve the difference measure between $E_{\psi }\left(x\right)$ and $D_{\phi }\left(E_{\psi }\left(x\right)\right)$ in Equation (5) based on the Wasserstein distance in the next section.

### 4.2. Priori Discriminative Loss of WDIM Method

When training the prior discriminator in the DIM approach, the main focus is on optimizing Equation (5) to train the model to satisfy the indistinguishability between the encoding vector $E_{\psi }\left(x\right)$ and the prior distribution *y*. In Section 3.2, we analyze in detail that the essence of the optimization Equation (5) is to learn the indistinguishability between the coding vector $E_{\psi }\left(x\right)$ and the prior distribution y using the idea of adversarial learning. At the same time, we also analyze that the adversarial learning prior discriminator is undesirable because the process of training the model is prone to “gradient explosion” and “gradient disappearance”, resulting in poor stability of the training model and thus difficulty in converging.

To solve the problem of “gradient explosion” and “gradient disappearance” of the DIM method, we need to construct a more stable objective function to guide the training of the prior discriminator and discard the loss function of the cross-entropy metric. Based on the superior performance of the Wasserstein distance measure of variability between any two distributions in Section 4.1, Equation (5) will be rewritten based on the Wasserstein distance in our WDIM method. Therefore, we propose the method based on the Wasserstein distance to approximate the distance between $E_{\psi }\left(x\right)$ and $D_{\phi }\left(x\right)$ in Equation (5), which is rewritten as follows:

(9) $${\left(\hat{\omega },\hat{\psi }\right)}_{P}=\underset{\psi ,\phi }{\arg\min}WDAA\left(D_{\phi }\left(y\right),D_{\phi }\left(E_{\psi }\left(x\right)\right)\right)$$

where the *x* samples are derived from the training data distribution P and the *y* samples are derived from the standard orthogonal distribution V.

The prior discriminant network in the WDIM method is trained by optimizing the objective function Equation (9), thus providing an effective loss value. Thus, the prior discriminator cannot distinguish whether the input comes from the coding vector $E_{\psi }\left(x\right)$ or from the *y* of the prior distribution, thus achieving that the output vector $E_{\psi }\left(x\right)$ of the encoder obeys the prior distribution as much as possible, and this more regular coding vector facilitates feature decoupling for learning of downstream tasks. Finally, the complete objective function of our WDIM method is given as follows:

(10) $$\begin{array}{cc} \;\mathrm{Loos}\;= & \underset{\omega_{1},\omega_{2},\omega_{3},\psi ,\phi ,\theta }{\arg\max}\left(\alpha {\hat{I}}_{\omega_{1},\psi }^{JSD}\left(X;E_{\psi }\left(X\right)\right)+\frac{\beta }{M^{2}}\sum_{i=1}^{M^{2}}{\hat{I}}_{\omega_{2},\psi }\left(X^{i};E_{\psi }\left(X\right)\right)\right)\\ \; & +\underset{\psi ,\phi }{\arg\min}\gamma WDAA\left(D_{\phi }\left(y\right),D_{\phi }\left(E_{\psi }\left(x\right)\right)\right) \end{array}$$

Compared with the full objective function Equation (10) of the DIM method, we only improve the calculation of the loss value of the prior discriminator part and do not add additional calculations. However, this improvement makes the training of the WDIM method more stable than that of the DIM method, without the problems of “gradient explosion” and “gradient disappearance”. The adversarial training allows the distribution of the coding vector $E_{\psi }\left(x\right)$ to converge consistently to the prior distribution V.

In Algorithm 2, we give the complete pseudo-code of the WDIM method for training the model, where the estimation of global mutual information and local mutual information is implemented using the “negative sampling” method. In the encoder network, we convolve the input data $x_{0}$ to obtain the intermediate feature *x*, and then pass *x* through the back part of the encoder network to obtain the coding vector $E_{\psi }\left(x\right)$. In the prior discriminator $D_{\phi }\left(\cdot \right)$, a batch of samples *y* with $E_{\psi }\left(x\right)$ is randomly sampled from the prior distribution as the input to $D_{\phi }\left(\cdot \right)$, and $D_{\phi }\left(y\right)$ and $D_{\phi }\left(E_{\psi }\left(x\right)\right)$ are output, respectively. When $D_{\phi }\left(y\right)\approx D_{\phi }\left(E_{\psi }\left(x\right)\right)$, we have reason to believe that the encoding vector $E_{\psi }\left(x\right)$ approximately obeys the prior distribution, so that $E_{\psi }\left(x\right)$ is indistinguishable from *y*. The core work of the WDIM method is to estimate the mutual information between the hidden layer *x* and the encoding vector $E_{\psi }\left(x\right)$. Therefore, in the global mutual information, estimator $T_{\psi ,\omega_{1}}\left(\cdot \right)$ first *x* is passed through a convolutional network that splices the spreading vector with $E_{\psi }\left(x\right)$ on the batch to obtain the positive sample pair $\left(x,E_{\psi }\left(x\right)\right)$. To obtain the negative sample pair $\left(x^{\prime },E_{\psi }\left(x\right)\right)$, we randomly disorder $x^{\prime }$ in batch *x*.
**Algorithm 2** WDIM algorithm**Input:** epochs, η, α, β, and γ. 1: $W\leftarrow \left\{\omega_{1},\omega_{2},\psi ,\phi \right\}\;$Combined parameters and random initialization. 2: i←1 3: **while** i<epochs **do** 4:     A batch of ${\left\{x_{0}^{i}\right\}}_{i=1}^{B}∼P_{\mathrm{data}}$, is sampled, and thus the intermediate features ${\left\{x^{i}\right\}}_{i=1}^{B},{\left\{y^{i}\right\}}_{i=1}^{B}∼N\left(0,1\right)$ of the encoder are obtained. Positive and negative sample pairs $\left(x^{i},E_{\psi }\left(x^{i}\right)\right)$ and $\left({\left(x^{i}\right)}^{\prime },E_{\psi }\left(x\right)\right)$ for estimating global mutual information are obtained by prediction processing. The positive and negative sample pairs $\left(C_{\psi }^{\left(i\right)},E_{\psi }\left(x^{i}\right)\right)$ and $\left({\left(C_{\psi }^{\left(i\right)}\right)}^{\prime },E_{\psi }^{\prime }\left(x^{i}\right)\right)$ of the estimated local mutual information are likewise obtained. 5:     ${\left(\hat{\phi },\hat{\psi }\right)}_{p}\leftarrow \frac{1}{B}\sum_{i=1}^{B}WDAA\left(D_{\phi }\left(y^{i}\right),D_{\phi }\left(E_{\psi }\left(x^{i}\right)\right)\right)$ 6:     ${\left({\hat{\omega }}_{1},\hat{\psi }\right)}_{G}\leftarrow \frac{1}{B}\sum_{i=1}^{B}\left[-sp\left(-T_{\psi ,\omega_{1}}\left(x^{i},E_{\psi }\left(x^{i}\right)\right)\right)\right]$$-\frac{1}{B}\sum_{i=1}^{B}\left[sp\left(T_{\psi ,\omega_{1}}\left({\left(x^{i}\right)}^{\prime },E_{\psi }\left(x^{i}\right)\right)\right)\right]$ 7:     ${\left({\hat{\omega }}_{2},\hat{\psi }\right)}_{L}\leftarrow \frac{1}{M^{2}}\sum_{i=1}^{M^{2}}\left[-sp\left(-T_{\psi ,\omega_{2}}\left(C_{\psi }^{\left(i\right)},E_{\psi }\left(x^{i}\right)\right)\right)\right]$$\;\;\;-\frac{1}{M^{2}}\sum_{i=1}^{M^{2}}\left[sp\left(T_{\psi ,\omega_{2}}\left({\left(C_{\psi }^{\left(i\right)}\right)}^{\prime },E_{\psi }\left(x^{i}\right)\right)\right)\right]$ 8:     Loss $\leftarrow \gamma {\left(\hat{\phi },\hat{\psi }\right)}_{p}-\left(\alpha {\left({\hat{\omega }}_{1},\hat{\psi }\right)}_{G}+\beta {\left({\hat{\omega }}_{2},\hat{\psi }\right)}_{L}\right)$ 9:     ∇W←∂ Loss /∂W            //Calculation gradients10:     W←W+ηAdam(W,∇W)//Update parameters11:     i←i+112: **end while**

Such processing is efficient and necessary for the “negative sampling” estimation method. In the local mutual information estimator $T_{\psi ,\omega_{2}}\left(\cdot \right)$, to estimate the local mutual information between *x* and $E_{\psi }\left(x\right)$, $E_{\psi }\left(x\right)$ is extended so that the extended $E_{\psi }\left(x\right)$ has the same dimensions as $C_{\psi }$, thus forming feature vectors of feature channel size at the points of the output feature matrix. Therefore, by using the chaotic order on the batch, the positive sample pair $\left(C_{\psi }^{\left(i\right)}\right.$, $\left.E_{\psi }\left(x\right)\right)$ and the negative sample pair $\left({\left(C_{\psi }^{\left(i\right)}\right)}^{\prime },E_{\psi }^{\prime }\left(x\right)\right)$ can be obtained to obtain the outputs $T_{\psi ,\omega_{2}}\left(C_{\psi }^{\left(i\right)},E_{\psi }\left(x\right)\right)$ and $T_{\psi ,\omega_{2}}\left({\left(C_{\psi }^{\left(i\right)}\right)}^{\prime },E_{\psi }\left(x\right)\right)$, respectively.

## 5. Experiments

To give comparative experiments with fairness, we choose PyTorch as the experimental platform, and uniformly give the same network model and hyper-parameter settings in the DIM method and WDIM method, except for the different algorithms.

### 5.1. Network Parameters

According to the architecture of our proposed WDIM method, as shown in Figure 1, we give the settings of each sub-network model parameter in the whole network architecture and the initialization parameter settings in the algorithm.

Setting of sub-network model parameters: the first sub-network responsible for feature extraction is the encoder network *E*. This sub-model is always present during the training process of combining the sub-network models, and in Table 1, we ignore the symbolic representation of this sub-network, which consists of four convolutional layers and one flatten layer, in the process of the representation of the abbreviated symbols. The second sub-network model is the a priori discriminative network *D*, which is responsible for restricting the output of the encoder network *E* to move closer to a positive-too distribution, making the output of the encoder network more regular. The third sub-network is the global mutual information maximization network model *G*. This network consists of two convolutional layers and two fully connected layers, which are used to limit the output of the second layer of encoder *E* to maximize the global mutual information between the output layers. The fourth sub-network is the local mutual information maximization network model *L*, which consists of three convolutional layers and is used to limit the local mutual information maximization between the output of the second layer of the encoder *E* and the output layer.

It should be noted that C in Table 1 denotes the classification network, which is designed after the whole network has been trained with the WDIM method. Taking WDIM(LGPC) as an example for the illustration of the network training process, the training data enter the feature extraction network to obtain the extracted feature vectors, and the loss value of the global mutual information maximization network G is calculated according to Equation (2). Similarly, the loss value of local mutual information maximization network L is calculated according to Equation (4). Calculate the loss value of the a priori discriminative network according to Equation (9), and finally train the whole network model by combining the networks E, G, L, and P according to Equation (10) to obtain the feature vectors, which at this time are obtained with unsupervised training. After obtaining the feature vectors, the feature vectors are used as inputs to the classification network C for the classification task. At this point, we can prove the effectiveness of the WDIM method for extracting features from the accuracy of the experiment.

Initialization parameter settings in the algorithm: in Algorithm 1, P(x) denotes the output of the classification network, i.e., the predicted value of the model, Q(y) denotes the labels of the training data, the maximum number of iterations max_iter=100, and the error threshold err_thresh=0.1. In Algorithm 2, the maximum number of iterations epochs=420, the batch size batch_size=64, learning rate η=0.001, loss weight α=0.5 for the global mutual information maximization network, β=1.0 for the local mutual information maximization network, and γ=0.1 for the a priori discriminative network.

We analyze the training stability of the models and the comparative experiments in the unsupervised classification scenario for the DIM and WDIM methods on three public datasets, CIFAR10, CIFAR100, and STL10.

### 5.2. Comparison of Model Training Stability

In Section 3.2, we quantitatively analyze the main reasons for the instability of the DIM method training model from a theoretical point of view and rewrite the optimization objective function Equation (5) of the DIM method based on the Wasserstein distance in Section 4.2, to obtain Equation (9). For improving the stable training of the model, the significance of such an improvement is derived from theoretical analysis. We have reason to believe that in our WDIM method, the training process of the prior discriminator does not result in the phenomenon of “gradient explosion”, and “gradient disappearance” does not occur in our WDIM method.

**Figure 4:**
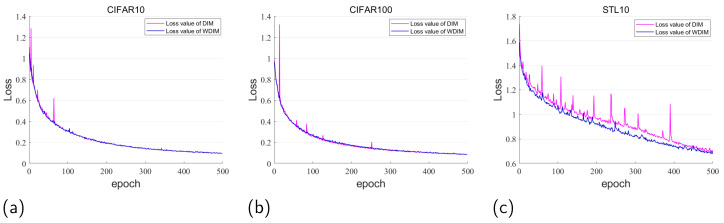
Comparison of the loss value curves of the DIM method and the WDIM method for training models on the datasets CIFAR10, CIFAR100, and STL10.

We compare the training models of the DIM method and WDIM method on CIFAR10, CIFAR100, and STL10 datasets, and find that the loss value of the training model of the DIM method always appears as “nan” when the batch_size<64, which leads to the phenomenon of the “gradient explosion” that we mentioned. Therefore, after repeating the experiment several times, we chose one successful training of the DIM method as a comparative experiment. As can be seen in Figure 4a, the loss value of the DIM method in the first 50 epochs on the public dataset CIFAR10 shows large fluctuations, but the training of the model still converges in the end. On the contrary, in our WDIM method, the loss value has been in a slowly decreasing state, and the model training is very stable and eventually performs as well as the DIM method. In addition, on the CIFAR100 dataset, as shown in Subfigure (b), the training of the DIM method still has some fluctuations, while our method still maintains a relatively stable training pattern, and it is obvious that our WDIM method maintains a highly significant level of training models. As can be seen in Subfigure (c), due to the small number of training samples in the STL10 dataset, in this case, the gradient direction found in the optimization process is somewhat different from the optimal gradient direction, so both methods have some fluctuations in the process of model training, but such fluctuations do not affect the final convergence of the model. The loss fluctuation calculated by our method is smaller and always smaller than the loss value of the DIM method. This fully reflects that the WDIM method is more capable of providing effective gradient training and faster convergence. In terms of overall performance, our method WDIM performs at least as well as the DIM method on the basis that the DIM method can train the model stably, and more importantly, the WDIM method consistently performs very well on the training model.

By comparing the stability of the trained models of the DIM method and the WDIM method on the CIFAR10, CIFAR100, and STL10 datasets, it can be found that the DIM method only supports the training process of large batches because, in the prior discriminator training of the DIM method using the adversarial training method there is a “gradient explosion” phenomenon. Large batches of training samples can alleviate the loss value tends to 0 or 1, and thus can stabilize the model training. Our WDIM method does not suffer from “gradient explosion” from the principle. The experiments show that the WDIM method performs as well as the DIM method under the conditions of stable training of the DIM method. Therefore, we can believe that the WDIM method is very significant and advantageous in the stability of the training model.

### 5.3. Comparative Accuracy of Unsupervised Learning Classification

We give the symbolic descriptions in Table 1, where “conv” denotes the output features of the last convolutional layer of the feature extraction network, “fc(1024)” denotes that the dimension of the feature vector output by the feature extraction network is 1024 dimensions, and “fc(64)” denotes that the dimension of the feature vector output by the feature extraction network is 64 dimensions. *L* is for the local mutual information maximization network, *G* is for the global mutual information maximization network, *P* is for a priori discriminant network, and *C* is for the classification network. For the downstream classification task, we give the comparative experiments of the DIM method and the WDIM method on the publicly available datasets CIFAR10, CIFAR100, and STL10, and the experimental results are shown in Table 1. Among them, the DIM method trains the DIM(G), DIM(DV), DIM(JSD), and DIM(infoNCE) models with the iteration step epoch = 1000, and our WDIM methods WDIM(GC), WDIM(LC), WDIM(GPC), WDIM(LPC), and WDIM(LGPC) models with iteration step epoch=420. From the iteration step epoch, our method requires fewer training iteration steps than the DIM method training iteration steps.

During the experiments, we first make sure that the DIM method can train the model stably, and then make a comparison. From the results of the classification experiments, our WDIM method has the advantage of faster convergence of the training model; however, the experimental accuracy of our proposed WDIM method is sometimes lower than that of the DIM method, and it is not always bad, and we analyze that the main reason may be due to the difference in the structure of the model, the different hyper-parameter settings, and other different reasons. During our experiments, the number of times we trained our model epoch = 420, is based on this number of training rounds, while the epoch used by the DIM method = 1000, as the conclusion of the model convergence in Figure 4. At the same time, we also further train our network model many times, and there will be about a 1–2.5% difference with DIM. It is not difficult to understand that there will always be some difference in each time of model training, e.g., the WDIM(GC) in the STL10 dataset is higher than that in the DIM method by 4.69%. But we feel that such a difference is objective.

## 6. Conclusions

In this paper, a DIM method based on mutual information maximization is studied in depth. It is found that the cross-entropy loss function used in this method suffers from the phenomena of “gradient explosion” and “gradient vanishing” during the training process, which is the root cause of unstable model training and slow model convergence. In this regard, the WDIM method based on Wasserstein distance is proposed to solve the above problems. The cross-entropy calculation loss is discarded in the WDIM method, and the distance between the output of the Wasserstein distance metric encoder and the prior distribution is used as the loss value for training the prior discriminant network. We have validated the unsupervised classification task on several public datasets, and the theoretical study and experimental results show that the proposed WDIM method is more stable in updating the model parameters and the model converges faster, among other advantages.

With the above theoretical studies, future thoughts extend the application of the WDIM method to datasets with deeper network models and more complex training datasets. Further thoughts to carry out feature decoupling in machine learning as well as feature non-interpretability using mutual information as a theoretical basis. The purpose is to provide a reference for researchers to re-conceptualize unsupervised interpretable machine learning, aiming to provide a research idea and research direction for the new generation of AI model training.

## Figures and Tables

**Table 1:** Comparison of DIM and WDIM methods for classification experiments on CIFAR10, CIFAR100, and STL10 datasets.

Model	CIFAR10			CIFAR100			STL10		
	conv	fc(1024)	fc(64)	conv	fc(1024)	fc(64)	conv	fc(1024)	fc(64)
DIM(G)	52.20%	52.84%	43.17%	27.68%	24.35%	19.98%	42.03%	30.82%	28.09%
DIM(DV)	72.66%	70.60%	64.71%	48.52%	44.44%	39.27%	69.15%	63.81%	61.92%
DIM(JSD)	73.25%	73.62%	66.96%	48.13%	45.92%	39.60%	72.86%	70.85%	65.93%
WDIM(GC)	53.42%	51.72%	42.89%	29.24%	24.88%	20.14%	46.72%	41.01%	36.46%
WDIM(LC)	72.22%	71.83%	65.26%	44.51%	44.12%	39.02%	68.47%	65.46%	62.56%
WDIM(GPC)	56.57%	54.89%	44.15%	31.18%	24.04%	19.08%	44.93%	39.93%	35.45%
WDIM(LPC)	72.21%	70.41%	65.89%	45.13%	44.76%	39.97%	66.51%	65.44%	64.66%
WDIM(LGPC)	70.67%	69.06%	64.71%	40.23%	39.06%	37.54%	63.33%	62.68%	61.23%

## Data Availability

Data are contained within the article.
